# Adult Prevalence of Epilepsy in Spain: EPIBERIA, a Population-Based Study

**DOI:** 10.1155/2015/602710

**Published:** 2015-12-10

**Authors:** Pedro J. Serrano-Castro, Jose Angel Mauri-Llerda, Francisco José Hernández-Ramos, Juan Carlos Sánchez-Alvarez, Beatriz Parejo-Carbonell, Pablo Quiroga-Subirana, Fernando Vázquez-Gutierrez, Sonia Santos-Lasaosa, Carolina Mendez-Lucena, Luis Redondo-Verge, Carlos Tejero-Juste, Clara Morandeira-Rivas, Jerónimo Sancho-Rieger, Jorge Matías-Guiu

**Affiliations:** ^1^Unidad de Neurología y Neurofisiología, Complejo Hospitalario Torrecárdenas, Calle Hermandad de Donantes de Sangre S/N, 04009 Almería, Spain; ^2^Servicio de Neurología, Hospital Clínico Universitario Lozano Blesa, Avenida San Juan Bosco 15, 50009 Zaragoza, Spain; ^3^Unidad de Neurología, Hospital de Llerena, Avenida Badajoz 1, 06900 Llerena, Badajoz, Spain; ^4^Servicio de Neurología, Hospital Clínico Universitario San Cecilio, Calle Dr. Oloriz 16, 18012 Granada, Spain; ^5^Servicio de Neurologia, Hospital Clínico San Carlos, Calle del Profesor Martín Lagos S/N, 28040 Madrid, Spain; ^6^Servicio de Neurología, Hospital Universitario Virgen Macarena, Avenida Doctor Fedriani 3, 41071 Sevilla, Spain; ^7^Servicio de Neurología, Hospital General Universitario de Valencia, Calle de la Casa Misericordia 12, 46014 Valencia, Spain

## Abstract

*Background*. This study assesses the lifetime and active prevalence of epilepsy in Spain in people older than 18 years.* Methods*. EPIBERIA is a population-based epidemiological study of epilepsy prevalence using data from three representative Spanish regions (health districts in Zaragoza, Almería, and Seville) between 2012 and 2013. The study consisted of two phases: screening and confirmation. Participants completed a previously validated questionnaire (EPIBERIA questionnaire) over the telephone.* Results*. A total of 1741 valid questionnaires were obtained, including 261 (14.99%) raising a suspicion of epilepsy. Of these suspected cases, 216 (82.75%) agreed to participate in phase 2. Of the phase 2 participants, 22 met the International League Against Epilepsy's diagnostic criteria for epilepsy. The estimated lifetime prevalence, adjusted by age and sex per 1,000 people, was 14.87 (95% CI: 9.8–21.9). Active prevalence was 5.79 (95% CI: 2.8–10.6). No significant age, sex, or regional differences in prevalence were detected.* Conclusions*. EPIBERIA provides the most accurate estimate of epilepsy prevalence in the Mediterranean region based on its original methodology and its adherence to ILAE recommendations. We highlight that the lifetime prevalence and inactive epilepsy prevalence figures observed here were compared to other epidemiological studies.

## 1. Introduction

Although epilepsy is the most frequent chronic neurological disease affecting all age groups, there is a lack of knowledge about its epidemiology in Europe [1]. While northern European studies of incidence and prevalence are relatively numerous, such studies remain scarce in central and southwest Europe. Furthermore, most of these studies were completed two or even three decades ago [2–5]. To our knowledge, only three recent and relevant population-based studies following International League against Epilepsy (ILAE) recommendations for epidemiological studies in epilepsy have been completed in the Mediterranean region; these studies examined three Italian municipalities, one suburb of a Turkish city (door-to-door studies in both cases), and one French city (not door-to-door) [6–8]. Active prevalence, defined as epileptic seizures in the last 5 years, ranged from 3.2 to 7.8 per 1,000 people [1]. In the case of Spain, several studies have been carried out to date. In 2001, Luengo et al. focused exclusively on the northeastern part of the Region of Madrid, and their study was based on hospital records [9]. Durá-Travé et al. published incidence data for epilepsy and epileptic syndromes among children in one Spanish region, Navarre, in 2008 [10]. In 2009, Benavente et al. quantified the prevalence of epilepsy among adolescents in the small city of Huesca [11]. A recent review by Garcia-Martin et al. included an epidemiological study of epilepsy in the city of Málaga, in southern Spain [12]. The methodological limitations present in each of these studies prevent their results from being extrapolated to the national level. Three are restricted to just one region (Madrid, Navarre, Huesca, or Málaga), two survey a pediatric or adolescent population [10, 11], and three are hospital-based studies [9, 11, 12]. Therefore, no accurate data reflecting the prevalence of epilepsy in Spain are currently available in the literature.

On the other hand, worldwide migratory flows have grown in last decade. Most immigrants to Spain are from South America, Asia, or Africa. The prevalence of epilepsy has been shown to depend on the country's healthcare system and socioeconomic status. In fact, reported prevalence from South America, Asia, or Africa is higher than data from European or North American countries [13]. For this reason, the epidemiology of epilepsy in southwestern Europe, and specifically in the Mediterranean region, should be revisited and updated. The objective of this study was to assess the lifetime and active prevalence of epilepsy in Spain by extrapolating results from three representative Spanish regions.

## 2. Material and Methods

**Figure 1:**
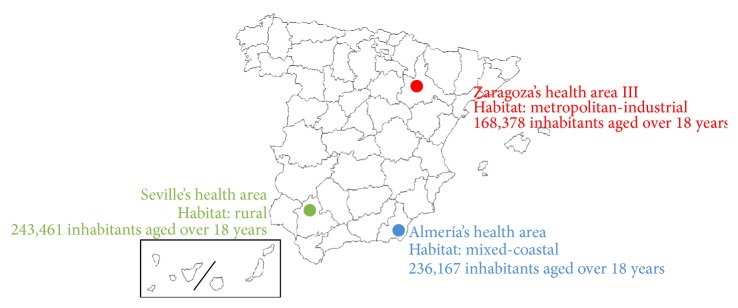
Location of the three representative Spanish regions.

EPIBERIA is an epidemiological study of epilepsy prevalence in Spain that uses a population-based model with denominator-controlled data taken from three representative Spanish regions between 2012 and 2013. Regions were as follows: (1) Zaragoza health district III (an urban industrial area), comprising a population of 168,378 inhabitants over 18 years old and represented by Hospital Clínico Universitario Lozano Blesa (Zaragoza, Spain); (2) an Almería health district (a mixed rural and urban coastal region), comprising a population of 236,167 inhabitants over 18 years old and represented by Complejo Hospitalario Torrecárdenas (Almería, Spain); and (3) a Seville health district (a rural area), with a population of 243,461 inhabitants over 18 years old and represented by Hospital Universitario Virgen Macarena (Seville, Spain). Population data were taken from the database of the National Statistics Institute of Spain (Instituto Nacional de Estadística, INE). These three regions were chosen based on their different sociodemographic and climatic characteristics in order to enable extrapolation of results to the national level. Whereas the gross domestic product (GDP) per capita in Almería and Seville (74.3% and 80.3%, resp.) is below the Spanish national average, Zaragoza level is 110.8% of Spain's GDP per capita (data from INE, 2012). There are no significant differences in educational level between residents of these regions. The literacy rate in 2012 in Spain was homogeneous and above 97% of the adult population. Climatic features of our three regions are heterogeneous and represent the wide variety of climates present in Spain. Zaragoza has a continental climate. Winters are cold, with night frosts and fogs, and summers are warm. The annual average precipitation is about 315 mm. Almería occupies the southeast corner of the Iberian Peninsula. It has a dry Mediterranean climate, with mild temperatures throughout the year. With an average of 2,965 hours of sunshine and 106 cloudless days a year, it is one of the sunniest parts of Europe. The third selected area, dominated by vast forests of oak trees, is a rural health district in Seville. It enjoys a Mediterranean climate with hot dry summers and mild winters. The average annual precipitation is 810 mm, with considerable seasonal variations. The location of the districts selected to represent Spain is shown in Figure 1. The overall target population was 648,016 inhabitants over 18 years old.

Table 1 shows the distribution of inhabitants by district and age. The proportion of inhabitants over 60 years old is higher in Zaragoza (33.8%) than in Almería (23.35%) or Seville (21.11%), reflecting a higher aging index. In Spain, the percentage of the total population over 60 years old is 27.54% (INE).

For all these demographic, geographic, and socioeconomic reasons we consider the population of the three selected regions is representative of the Spanish population.

All procedures were performed in accordance with guidelines established by the Declaration of Helsinki and Spanish laws. The ethics committee at Hospital Torrecárdenas (Almería) approved the study.

### 2.1. Source of the Sample

We requested a list of randomly selected candidates aged 18 and older in each specific health district, taken from the database listing all holders of Spanish public health cards in each of the three health districts studied. Proportions within each sample were required to reflect the population pyramid (sex and age) for the specified health district. A computer-based random number generator created the randomized list.

It should be clarified that Spain has a public healthcare system, which means that all individuals have the right to receive a health card free of charge. The administrative data linked to a single Spanish health card includes at least one personal telephone number. This telephone number can be a landline or a mobile and it identifies a single cardholder.

### 2.2. Determination of the Sample Size and Randomization

According to Picot et al. [8], the age-adjusted prevalence of active epilepsy was 5.4 per 1,000 in a French population (95% CI, 4.7–6.0); we used this data to estimate our sample size. The total population of our three selected areas is 648,016 inhabitants over 18 years old. When attempting to estimate prevalence in very large populations for low-prevalence diseases (as in this case), it is necessary to limit the sample size such that it permits a face-to-face survey. For this reason, the study design for EPIBERIA included determining 5% of the approximate population of one of our target provinces (approx. 240,000 inhabitants). This calculation delivered a final sample size of 12,000 inhabitants. Assuming this sample size, and a precision of 2%, we estimated that 492 valid surveys would be needed for each region. To allow for a 10% dropout rate in the confirmation phase, the minimum number of valid surveys required for the screening phase was raised to 541 per district. Assuming a 20% response rate, the total randomized sample size was established at 3,000 per district.

### 2.3. Experimental Phases

The EPIBERIA study consisted of two phases: screening and confirmation.

#### 2.3.1. Screening Phase

According to our calculated sample size, 3,000 participants over 18 years old were randomly selected from each district's database.

Within this selected population, 600 individuals were randomly selected to participate in the survey and 2,400 were identified as substitutes in case any selected candidates declined to participate. Participants completed the questionnaire over the telephone. The telephone survey was carried out by trained interviewers who had undergone specific training, but who had no knowledge of epilepsy and related conditions. Interviewers called up to three different times to reach the selected candidate. If candidates could not be contacted or declined to participate, the interviewer then called a substitute candidate. Substitutes were persons randomly extracted from the same local database for population that had been previously selected and for that our study population were representatives. The EPIBERIA questionnaire, which has been previously validated and published in Spanish [14], is based on the questionnaire validated by Ottman et al. for studying epilepsy epidemiology [15]. All individuals verbally gave informed consent to participate in the study since surveys of the screening phase were conducted by phone. It was properly reflected in the EPIBERIA questionnaire of each individual. The ethics committee approved this consent procedure.

In the screening phase, researchers selected any participants suspected of developing epilepsy, or of having developed the disease at any point during their lifetimes. To avoid selection bias, subjects were flagged as suspected epilepsy cases when they answered “yes” or “possible” to question number two (“[Other than the seizure[s] you had because of a high fever] Have you ever had, or has anyone ever told you that you had, a seizure disorder or epilepsy?”) or when they answered “No” to question number two but “yes” or “possible” to any item (A to G) on question number three (“[Other than the seizure[s] you had because of a high fever] Have you ever had, or has anyone ever told you that you had, any of the following…”) [14, 15]. Responses of “yes” or “possible” to either question ensured 100% sensitivity for identifying epilepsy among participants.

#### 2.3.2. A Confirmation Phase

Subjects suspected of developing epilepsy were asked to attend a face-to-face interview conducted by an experienced neurologist at their habitual healthcare centers. Subjects who declined to visit their healthcare centers were given the option of completing the interview by telephone. In both cases, the neurologist explored the subject's medical history and used such methods as EEG or magnetic resonance imaging (MRI) as needed to diagnose or rule out epilepsy. Epilepsy was diagnosed according to the 1993 ILAE criteria, that is, at least two unprovoked epileptic seizures, occurring more than 24 hours apart, in the patient's lifetime [16]. We chose this definition of epilepsy to enable comparisons with previous epidemiological studies. For the same reason, we also assume the definitions of cryptogenic and idiopathic epilepsy established by the ILAE in 1989 [17].

Confirmed cases of epilepsy were subsequently evaluated by EPIBERIA's Scientific Committee to corroborate the diagnosis and accuracy of the data.

### 2.4. Quantification of Epilepsy and Statistical Analysis

Valid questionnaires, confirmed by experienced neurologists and corroborated by main researchers, provided the data used to assess lifetime and active prevalence of epilepsy among participants. Estimated prevalence was calculated taking into account those individuals who did not participate in phase 2, by assuming that these participants had similar characteristics, and therefore similar rates, to those who did attend the confirmation phase. Active epilepsy rate, defined as the occurrence of one seizure in the previous five years, was also calculated [18]. Lifetime prevalence of epilepsy was calculated as crude prevalence and also standardized by age and sex to the European Standard Population [19]. Standardization was performed with Epidat software, version 4.0 (Xunta de Galicia, Spain). Data are expressed as percentages and 95% confidence intervals (95% CI), or as means and standard deviations (SD). We calculate exact CI considering a Poisson distribution. Statistical significance was established at *p* < 0.05. All statistical procedures were performed with SPSS 21.0 (IBM, Chicago, USA).

## 3. Results

**Figure 2:**
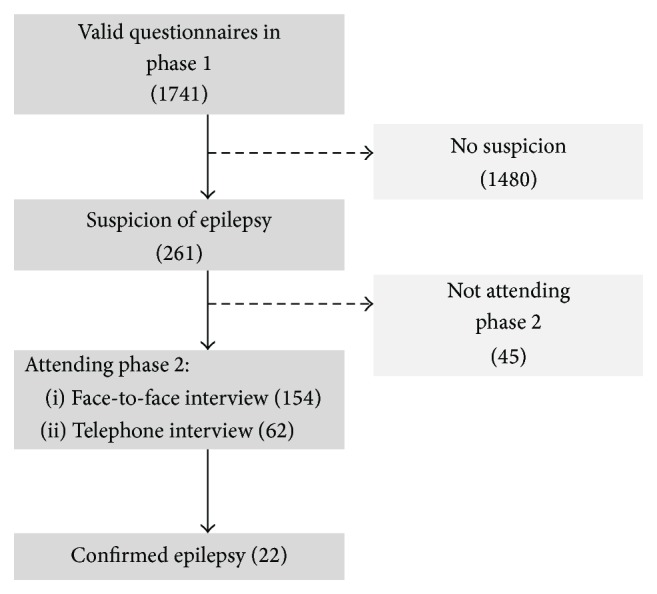
Flowchart of individuals participating in the study.

A total of 3,876 telephone calls were made in the screening phase; 3,175 calls were answered and 701 were not. The flowchart of individuals participating in the study is shown in Figure 2. Of the individuals who answered the phone call, 1,741 agreed to participate in the study. Although the response rate was 54.8%, 100% of the quota of randomized subjects required by the study design was met because nonresponders were replaced by previously selected alternates who were representative of study population, following the criteria explained in Section 2.

The valid questionnaires included 261 cases of suspected epilepsy (14.99%), homogeneously distributed across the three districts located in Zaragoza (13.12%), Almería (16.67%), and Seville (15.00%). The remaining 1,480 subjects were free of suspicion of epilepsy. Most of the 261 subjects with suspected epilepsy (82.75%) agreed to participate in phase 2 of the study; 154 completed the personal face-to-face interview and 62 completed a telephone interview. A total of 45 participants (17.25%) therefore dropped out of the study. Reasons why these individuals were missing in phase 2 were as follows: no answer or voice mail answer to the telephone call (25 individuals); having changed the address (2) or telephone number (2); call answered by a relative with cognitive impairment (2) or with no information about the person with suspected epilepsy (25); or refusal to participate (4). The total refusal rate was 1.53% of the total subjects with suspected epilepsy (261) and 0.22% of the total participants in phase 1 (1741). Individuals who completed phase 2 were distributed as follows: Zaragoza: 57 participants (80.28%); Almería: 93 participants (93.00%); and Seville: 66 participants (73.33%). After the phase 2 interview, a diagnosis of epilepsy was ruled out for 190 cases. A series of diagnostic tests (EEG and MRI) was required in 4 cases, but epilepsy was ruled out in all of them. A total of 22 participants fulfilled the ILAE diagnostic criteria for epilepsy.

Lifetime and active prevalence for each of the three representative regions are shown in Table 2.

The estimated mean lifetime prevalence, adjusted by age and sex per 1,000 people, was 14.87 (95% CI: 9.8–21.9). The estimated mean active prevalence, adjusted by age and sex per 1,000 people, was 5.79 (95% CI: 2.8–10.6). Broken down by sex, lifetime prevalence was 17.53 (95% CI: 10.6–28.3) in women and 12.44 (95% CI: 7.9–19.1) in men.

Table 3 shows us the lifetime prevalence broken down by age.

More detailed information relating to age, sex, and geographic distribution will be available (S1: distribution of subjects who agreed to participate in the study by age, sex, and geographic district; S2: distribution of subjects with suspicion of epilepsy by age, sex, and geographic district; and S3: distribution of subjects with suspicion of epilepsy who attended phase 2 interviews by age, sex, and geographic district, in Supplementary Material available online at http://dx.doi.org/10.1155/2015/602710).

### 3.1. Characteristics of Epileptic Subjects

The mean age (SD) was 44.13 (17.72) years. Mean age at onset of epileptic symptoms was 18.86 (16.50) years. Epileptic subjects comprised 14 women (63.6%) and 8 men (36.4%). Thirteen subjects (59.1%) experienced partial seizures with or without secondary generalization, eight (36.4%) had generalized tonic-clonic seizures, two (9.0%) had myoclonic seizures, and one (4.5%) experienced absence seizures. Broken down by etiology, there were eleven cases (50.0%) of symptomatic epilepsies, eight cases (36.4%) of idiopathic epilepsy, and three cases (13.6%) of cryptogenic epilepsy. A total of 13 subjects (59.1%) were not taking antiepileptic drugs (AEDs) at the time of the phase two interview. Therefore, the prevalence of inactive, untreated epilepsy was 7.46 per 1,000 people (95% CI: 3.10–11.80). The remaining nine patients (40.9%) were treated with AEDs in monotherapy, mainly valproic acid (four patients) and levetiracetam (two patients). The total number of active epilepsies we detected in the study was 8 patients (4 from Seville, 2 from Almería, and 2 from Zaragoza). More detailed demographic and clinical characteristics of the epileptic participants are available (S4: demographic and clinical characteristics of the epileptic participants).

## 4. Discussion

Since the ILAE's guidelines for epidemiological studies on epilepsy were published, few population-based studies have been performed in the Mediterranean region. Furthermore, very few studies have been conducted with methodologies similar to that of EPIBERIA. A door-to-door survey carried out in three Sicilian municipalities revealed an overall prevalence of active epilepsy of 3.3 per 1,000 people (3.5 for men and 3.2 for women) [6]. A study in a medium-sized French city (Béziers) showed an age-adjusted prevalence of active epilepsy of 5.4, higher in males (7.8) than females (5.2). The prevalence for epilepsy in remission with treatment was 0.7 [8]. Lastly, another door-to-door survey carried out in the Kucukcekmece region of Istanbul (Turkey) showed a lifetime prevalence of 8 per 1,000 people [7]. All three studies shared the methodological limitations associated with small sample sizes. To date, no information has been published about the prevalence of epilepsy among adults in Spain. Our study yielded a lifetime prevalence of 14.87 cases per 1,000 people over 18 years: 5.79 cases of active epilepsy and 9.08 cases of inactive epilepsy.

The active prevalence was in accordance with European mean values [1]. However, lifetime prevalence in our study is more comparable to figures obtained in studies in developing countries and rural settings, estimated at 15.4 cases per 1,000 people in the meta-analysis published in 2010 by Ngugi et al. [20]. There are some exceptions, such as the article by Kobau et al. (2004), which estimated a lifetime epilepsy prevalence of 2.1% in the states of Tennessee and Georgia in 2002 [21]. In any case, the methodology used in these studies differed from that of EPIBERIA. Although the reasons for our findings are probably multifactorial, they may reflect the changes in the social and health conditions in Spain in the last 50 years. Furthermore, we found no differences between our study and European studies completed before the increase in immigration from Africa, Asia, and South America.

Although data were not statistically significant, the surveyed districts showed a tendency toward differences in prevalence. The urban district in Zaragoza, characterized by a higher socioeconomic status and an older population, showed the highest prevalence. The lowest value was found in the mixed rural and urban coastal district in Almería.

No significant differences in prevalence were found for age or sex. The data for lifetime prevalence across all age groups could suggest a certain degree of poor recall. Otherwise, since the number of positive cases increases as people age, we would expect higher lifetime prevalence in older age groups.

The overall clinical characteristics of patients identified in our study are similar to what we anticipated. As such, partial seizures and symptomatic or cryptogenic epilepsies predominate over other seizure types or etiologies. We found a high percentage of patients with inactive epilepsy without treatment (59.1%). Although our study design does not allow us to draw conclusions from this data, it provides an opportunity for future research on the prognosis of epilepsy.

The value of our study resides in its novel methodological approach. First, we used the database of health cardholders registered with the Spanish public health system. This database provides several advantages compared to other more traditional data sources, such as the municipal census or telephone directories. For example, it can be used in populations distributed over larger areas and data can be filtered easily using computer software, as we have done here. In addition, health card data reflect the real population since individuals need their cards in order to have access to medical care. In contrast, census information is less reliable: even within the same region, an individual may not live in the town that he or she declares for official purposes. The Spanish public health system's user databases can also be used to generate automatic lists for use in surveys.

Second, the EPIBERIA study was conducted across three large regions with different demographical and socioeconomic characteristics. Since prevalence may be biased by local factors, the selection of representative regions with distinct features enables extrapolation of our results to the national level. Secondly, the two-phase approach chosen for the study is the most adequate for use in descriptive studies of low-prevalence diseases. Thirdly, recall and selection biases are common in this kind of studies; the potential for these two biases was minimized by adhering to the definition of epilepsy as a chronic disease and by the design itself, respectively. The strict criteria established for detecting epilepsy helped deliver accurate prevalence estimates for lifetime and active epilepsy. Moreover, the acceptable response rate in phase 1 (54.8%), the low refusal rate in phase 2 (<3.0%), and adjustment to European standard values determined the maximum degree of accuracy and permit extrapolation of the study to the western European population in general. Our active prevalence (5.79%) was slightly higher than the prevalence in the French study (5.4%) used to determine our sample size. This agreement in rates indicated a lack of type I and II errors in our data, thus validating and corroborating the results. The refusal rate in phase 2 was 1.53% of the total subjects with suspicion of epilepsy (261) and 0.22% of the total participants in phase 1 (1741). We concur that this small subgroup may differ from the group that initially agreed to participate in the study but we did not find why this would be true for the other groups of patients not attending phase 2 (e.g., phone call not answered or call answered by a relative with no information about the subject with suspected epilepsy).

Several limitations of our study should be discussed. The first is the use of a telephone survey method in the screening phase. Despite the existence of randomized comparative studies of response rate (RR) or effectiveness in field surveys, the information from the published literature reflects a variety of methods: different studies used telephone [22], e-mail, interactive voice response [23], or web-based surveys [24]. Nevertheless, most studies indicate that telephone surveys achieve the highest RRs [25], even more than mailed surveys [26]. Although some studies indicate that a high prevalence of nonresponses may bias the evaluation of results [27, 28], most authors consider that it does not influence results in field surveys [29]. Our study was designed with a structured telephone-based model in which the questionnaire was presented by a trained interviewer in such a way as to maximize the RR. In contrast with our estimate of 20% RR, the observed RR was 44.91%. This rate was higher than other rates reported in the literature [30], which indicated that the risk of a selection bias was lower than in the predominantly door-to-door epidemiological studies described in the literature. Our group has previously published a more detailed analysis of factors influencing response rates in this study [31]. Therefore, data from this study are suitable for making strong conclusions that will increase the effectiveness of future epidemiological studies.

Another possible limitation of our study is the existence of a recall bias that may have slightly underestimated our lifetime prevalence data, since all interviews were conducted in adult patients. We try to minimize this bias insisting that interviewers ask about existence of criteria for epilepsy “at any time in their life.” In any case, as has been discussed above, since the lifetime prevalence of epilepsy is a cumulative rate, the absence of growth in older ages in our study suggests a recall bias and therefore a tendency to underestimate our results.

Finally, our data on the prevalence of active epilepsy are only applicable to adult population, making them not comparable to those of other studies that include also child population. This should be considered a limitation of the study.

Many experts hypothesize that lifetime prevalence of epilepsy is underestimated in most epidemiologic studies (including door-to-door studies) due to the high early mortality rates in this disease, which results in prevalence being lower than the cumulative incidence rates. This tendency is more evident in lower income countries where patients have less access to antiepileptic medication and higher rates of infection-related epilepsies that are associated with high mortality [32]. Nevertheless, in EPIBERIA, the difference between real and observed prevalence is likely to be less pronounced since Spain's public healthcare system guarantees universal access to antiepileptic drugs and appropriate care for central nervous system infections.

## 5. Conclusions

EPIBERIA provides the most accurate estimate of epilepsy prevalence in the Mediterranean region due to its original methodology and adherence to ILAE recommendations. The lifetime prevalence was 14.87 cases per 1,000 people aged 18 years and over: 5.79 cases of active epilepsy and 9.08 cases of inactive epilepsy. The main contribution of our study is that it highlights high lifetime epilepsy and inactive epilepsy prevalence compared with other epidemiologic studies.

## Supplementary Material

Supplementary Material show us a more detailed information relating to age, sex, and geographic distribution of our sample.

## Figures and Tables

**Table 1:** Distribution of population by district and age.

	18–39 years	40–59 years	≥60 years	Total	Proportion of people over 60 years
Almería health district	99,617	81,403	55,157	236,177	23.35%
Zaragoza health district	57,042	54,411	56,925	168,378	33.80%
Seville health district	92,063	99,991	51,407	243,461	21.11%
Total Spanish population	14,642,820	13,544,712	10,715,484	38,903,016	27.54%

**Table 2:** Lifetime and active prevalence in the three representative regions.

	Total identified cases	Calculated mean crude prevalence per 1,000 people (95% CI)	Estimated cases in confirmation phase	Estimated mean crude prevalence per 1,000 people (95% CI)	Calculated mean overall prevalence, adjusted by age and sex per 1,000 people (95% CI)	Estimated mean overall prevalence, adjusted by age and sex per 1,000 people, corrected for dropouts (95% CI)
Zaragoza health district	7	12.94 (5.2–26.7)	8.71	16.11 (7.6–31.6)	12.30 (7.5–18.4)	14.87 (9.8–21.9)
Almería health district	7	11.66 (4.7–25)	7.52	12.54 (5.8–26.3)		
Seville health district	8	13.33 (5.8–26.3)	10.90	18.18 (9.2–32.8)		
Lifetime prevalence	22	12.63 (8–19.4)	26.58	15.27 (9.9–22.1)		

Active prevalence	8	4.59 (2–9.1)	9.66	5.55 (2.8–10.6)	4.80 (2–8.1)	5.79 (2.8–10.6)

**Table 3:** Lifetime prevalence in the three representative districts broken down by sex and age.

	Total obtained cases	Mean crude prevalence per 1000 people (95% CI)	Estimated cases in confirmation phase	Calculated mean overall prevalence per 1,000 people, adjusted by age and sex (95% CI)	Estimated mean overall prevalence per 1000 people, adjusted by age and sex, corrected for dropouts (95% CI)
Women	14	13.91 (7.6–23.3)	17.37	14.13 (7.6–23.3)	17.53 (10.6–28.3)
Men	8	10.87 (4.7–21.4)	9.14	10.27 (3.8–19.6)	12.44 (7.9–19.1)
Age 18–39 years	9	12.28 (5.6–23.3)	11.42	13.07 (6.5–25.1)	16.58 (6.6–26.5)
Age 40–59 years	8	12.06 (5.5–24.9)	9.34	10.58 (4.4–22.8)	12.36 (5.2–25.5)
Age ≥ 60 years	5	14.45 (4.7–33.8)	5.75	13.44 (3.2–29.7)	15.45 (5.0–32.9)
