# Supplementary material for: Adult Prevalence of Epilepsy in Spain: EPIBERIA, a Population-Based Study
Source: ScientificWorldJournal. 2015 Dec 10;2015:602710. doi: 10.1155/2015/602710 (PMC4689975; doi:10.1155/2015/602710)
Supplement: Supplementary file 1 — Supplementary Material show us a more detailed information relating to age, sex, and geographic distribution of our sample. [file 602710.f1.pdf]

S1. Distribution of subjects who agreed to participate in the study by age, sex, and geographic district

	Zaragoza		Almería		Seville		
**Age (years)**	**Men**	**Women**	**Men**	**Women**	**Men**	**Women**	Total
18-19	4	5	7	7	7	9	39
20-24	20	25	25	21	24	22	137
25-29	22	32	16	30	28	27	155
30-34	29	39	21	31	30	43	193
35-39	28	51	36	26	33	35	209
40-44	17	42	30	32	26	31	178
45-49	29	44	26	43	21	37	200
50-54	20	41	22	28	13	37	161
55-59	22	31	20	16	17	18	124
60-64	16	19	19	18	16	19	107
65-69	0	3	14	19	22	29	87
70-74	1	0	14	14	3	16	48
75-79	1	0	16	15	9	8	49
80-84	0	0	3	21	3	9	36
85-89	0	0	1	8	3	4	16
90-94	0	0	0	1	1	0	2
≥95	0	0	0	0	0	0	0
Total	209	332	270	330	256	344	1741

S2. Distribution of subjects with suspicion of epilepsy by age, sex, and geographic district

	**Zaragoza**		**Almería**		**Seville**		
**Age (years)**	**Men**	**Women**	**Men**	**Women**	**Men**	**Women**	Total
18-19	0	3	1	1	1	1	7
20-24	4	4	7	6	5	4	30
25-29	5	3	5	4	2	8	27
30-34	1	2	3	7	2	8	23
35-39	1	6	12	3	4	5	31
40-44	2	10	5	4	4	5	30
45-49	3	6	5	4	2	9	29
50-54	0	7	1	2	3	7	20
55-59	4	5	1	5	1	2	18
60-64	1	3	2	2	0	2	10
65-69	0	1	1	5	2	3	12
70-74	0	0	1	3	2	4	10
75-79	0	0	3	0	0	2	5
80-84	0	0	0	6	0	1	7
85-89	0	0	0	1	0	1	2
90-94	0	0	0	0	0	0	0
≥95	0	0	0	0	0	0	0
Total	21	50	47	53	28	62	261

S3. Distribution of subjects with suspicion of epilepsy who attended phase 2 interviews by age, sex, and geographic district

	**Zaragoza**		**Almería**		**Seville**		
**Age (years)**	**Men**	**Women**	**Men**	**Women**	**Men**	**Women**	Total
18-19	0	1	0	1	1	1	4
20-24	4	3	7	6	3	4	27
25-29	4	3	5	3	2	6	23
30-34	1	2	2	5	2	3	15
35-39	0	5	12	2	3	2	24
40-44	1	9	5	3	3	5	26
45-49	3	4	5	4	2	7	25
50-54	0	5	1	2	1	7	16
55-59	4	4	1	5	1	1	16
60-64	1	3	2	2	0	2	10
65-69	0	0	1	5	2	3	11
70-74	0	0	1	3	1	3	8
75-79	0	0	3	0	0	1	4
80-84	0	0	0	6	0	0	6
85-89	0	0	0	1	0	0	1
90-94	0	0	0	0	0	0	0
≥95	0	0	0	0	0	0	0
Total	18	39	45	48	21	45	216

S4 Demographic and clinical characteristics of the epileptic participants

ID	Age (years)	**Sex**	**Age at diagnosis (years)**	**Health Area**	**Tipo de crisis (ILAE)**	**Etiology**	**Active epilepsy**	**Comorbidity**	**Pharmacological Treatment**
**1**	82	F	40	Almeria	GTCS	Idiopathic	No	Cardiologic (pacemaker)	No EADs
**2**	65	M	13	Almeria	PS, PSSG	Cryptogenic	No	Dislipemia	No EADs
**3**	53	F	9	Zaragoza	PS	Idiopathic	No	No	No EADs
**4**	47	M	9	Almeria	PS, PSSG	Symptomatic (vascular)	No	Ischemic cardiopathy	No EADs
**5**	67	F	62	Seville	PS	Symptomatic (AVM)	Yes	Vascular pathology, Arthrosis	LEV
**6**	43	F	20	Zaragoza	PS, PSSG	Cryptogenic	No	Venous insufficiency	No EADs
**7**	24	F	4	Zaragoza	PS, PSSG	Symptomatic (post-encephalic)	Yes	No	VPA
**8**	32	F	20	Zaragoza	PS, PSSG	Symptomatic (left HS)	Yes	No	LTG
**9**	77	F	0	Seville	PS, PSSG	Symptomatic (connatal)	Yes	Mental retardation, Bilateral cataracts	No EADs
**10**	34	F	10	Seville	GTCS	Idiopathic	No	No	No EADs
**11**	26	M	1	Almeria	AS	Idiopathic	No	Headache	No EADs
**12**	25	F	16	Zaragoza	MS, CTCG	Idiopathic	No	No	LEV

**13**	48	F	32	Zaragoza	MS, CTCG	Idiopathic	No	No	VPA
**14**	20	F	10	Almeria	PS, PSSG	Symptomatic (posttraumatic)	No	Paroxysmal positional vertigo	No EADs
**15**	29	M	10	Almeria	CTCG	Symptomatic (vascular)	Yes	Vascular encephalopathy	No EADs
**16**	39	M	38	Almeria	GTCS	Symptomatic	Yes	Psychiatric (personality disorder)	TPM
**17**	40	F	39	Seville	PSSG	Symptomatic (AVM)	Yes	Iron deficiency anemia	CBZ
**18**	48	M	42	Seville	PS, PSSG	Symptomatic (posttraumatic)	Yes	HIV+, Hepatitis C, deep vein thrombosis	VPA
**19**	25	M	0	Seville	PS, PSSG	Symptomatic (connatal)	Yes	No	No EADs
**20**	53	F	10	Seville	PSSG	Cryptogenic	No	Depressive dysthymia	No EADs
**21**	34	M	8	Seville	GTCS	Idiopathic	No	No	VPA
**22**	60	F	22	Zaragoza	GTCS	Idiopathic	No	Depressive dysthymia, migraine, herniated disk	No EADs

F, Female; M, male; GTCS, generalized tonic-clonic seizure; PS, partial seizure; PSSG, partial seizure with secondary generalization; AS, absence seizure; MS, myoclonic seizure; AVM, arteriovenous malformation; HS, hippocampal sclerosis; AEDs, antiepileptic drugs; LEV, Levetiracetam; VPA, Valproic acid; LTG, Lamotrigine; TPM, Topiramate; CBZ, Carbamazepine
